# Description of a Multi-faceted COVID-19 Pandemic Physician Workforce Plan at a Multi-site Academic Health System

**DOI:** 10.1007/s11606-020-06543-1

**Published:** 2021-02-09

**Authors:** Sachin R. Pendharkar, Evan Minty, Caley B. Shukalek, Brendan Kerr, Paul MacMullan, Parabhdeep Lail, Kim Cheema, Nimira Alimohamed, Thomas Allen, Meghan E. O. Vlasschaert, Rahim Kachra, Irene W. Y. Ma, Kerri A. Johannson, Paul S. Gibson, Meghan J. Elliott, Adam Papini, Stephanie Smith, Jane Lemaire, Shannon M. Ruzycki, Angela Hunter, Wendy Desjardins-Kallar, Jeffrey P. Schaefer, Kelly B. Zarnke, Aleem Bharwani, Gabriel E. Fabreau

**Affiliations:** 1 Department of Medicine, Cumming School of Medicine, University of Calgary, Foothills Medical Centre – North Tower, Calgary, AB Canada; 2 Department of Community Health Sciences, Cumming School of Medicine, University of Calgary, Calgary, AB Canada; 3 O’Brien Institute for Public Health, Cumming School of Medicine, University of Calgary, Calgary, AB Canada; 4 Cumming School of Medicine, University of Calgary, Health Sciences Centre, Calgary, AB Canada

**Keywords:** COVID-19, health workforce, disease outbreaks, delivery of healthcare

## Abstract

**Background:**

The evolving COVID-19 pandemic has and continues to present a threat to health system capacity. Rapidly expanding an existing acute care physician workforce is critical to pandemic response planning in large urban academic health systems.

**Intervention:**

The Medical Emergency-Pandemic Operations Command (MEOC)—a multi-specialty team of physicians, operational leaders, and support staff within an academic Department of Medicine in Calgary, Canada—partnered with its provincial health system to rapidly develop a comprehensive, scalable pandemic physician workforce plan for non-ventilated inpatients with COVID-19 across multiple hospitals. The MEOC Pandemic Plan comprised seven components, each with unique structure and processes.

**Methods:**

In this manuscript, we describe MEOC’s Pandemic Plan that was designed and implemented from March to May 2020 and re-escalated in October 2020. We report on the plan’s structure and process, early implementation outcomes, and unforeseen challenges. Data sources included MEOC documents, health system, public health, and physician engagement implementation data.

**Key Results:**

From March 5 to October 26, 2020, 427 patients were admitted to COVID-19 units in Calgary hospitals. In the initial implementation period (March–May 2020), MEOC communications reached over 2500 physicians, leading to 1446 physicians volunteering to provide care on COVID-19 units. Of these, 234 physicians signed up for hospital shifts, and 227 physicians received in-person personal protective equipment simulation training. Ninety-three physicians were deployed on COVID-19 units at four large acute care hospitals. The resurgence of cases in September 2020 has prompted re-escalation including re-activation of COVID-19 units.

**Conclusions:**

MEOC leveraged an academic health system partnership to rapidly design, implement, and refine a comprehensive, scalable COVID-19 acute care physician workforce plan whose components are readily applicable across jurisdictions or healthcare crises. This description may guide other institutions responding to COVID-19 and future health emergencies.

**Supplementary Information:**

The online version contains supplementary material available at 10.1007/s11606-020-06543-1.

## INTRODUCTION

In 2020, the COVID-19 pandemic has had far-reaching impacts on health, society, and the global economy. In Canada, as of November 1, 2020, there have been over 240,000 cases, leading to 10,000 deaths and prompting many Canadian provincial governments to declare public health emergencies.^1^ Early in the pandemic, limited evidence about COVID-19 transmission and clinical characteristics combined with rapid case transmission, early projections suggesting exponential growth, and emerging reports from severely affected jurisdictions such as Lombardy, Italy, and New York City, USA, prompted concerns in many jurisdictions that the number of COVID-19-infected patients would exceed healthcare system capacity.

In March 2020, epidemiologic data revealed that a disproportionate number of cases were occurring in Alberta’s most populous city, Calgary (population 1.5 million).^2^ Demand projections based upon COVID-19 case doubling times (3–4 days) for the Calgary Zone^3^ indicated the existing acute care physician workforce would be overwhelmed, without a clear timeline for onset or trajectory. Scientific understanding of COVID-19 was rapidly evolving; however, there was limited evidence to guide the development and implementation of a rapidly scalable acute care physician workforce plan. The Department of Medicine (DOM) at the University of Calgary and Alberta Health Services (AHS)—Calgary Zone established the Medical Emergency-Pandemic Operations Command (MEOC) to rapidly design, test, and implement a physician workforce plan for hospitalized, non-ventilated inpatients with COVID-19.

In this manuscript, we describe MEOC’s multi-faceted pandemic physician workforce plan from development through early implementation to current re-escalation. We aim to provide timely guidance to other jurisdictions facing similar physician workforce planning challenges amidst the COVID-19 pandemic, as well as for future health crises.

## METHODS

In this manuscript, we describe the MEOC Pandemic Plan from March 20 to October 26, 2020, using Donabedian’s Health Services Quality framework to investigate the structure, process, and implementation outcomes of the plan’s seven key components.^4, 5^ We also discuss unforeseen challenges in development and implementation. Data sources include MEOC team documents, clinical algorithms, and process descriptions that were designed in advance, or in response to formal and informal stakeholder feedback during implementation. The dynamic description of implementation, de-escalation, and re-escalation of the pandemic plan is mapped against hospital demand and COVID-19 case volume from March 5 to October 26, 2020, using publicly reported provincial health data^2^ and provincial health system operational data for COVID-19 hospitalizations. This report follows the Revised Standards for Quality Improvement Reporting Excellence (SQUIRE 2.0).^6^ The Institutional Research Ethics Board provided an exemption from formal approval.

## CONTEXT

Alberta has a publicly funded health system with hospitals and many ambulatory services coordinated by a single provincial health authority (AHS). There are five geographic zones within AHS that manage local clinical operations, supply chain management, and other health system functions. The Calgary Zone comprises 14 hospitals, including four large quaternary and tertiary care urban adult and one tertiary-care pediatric hospital, that together contain 2791 hospital beds for approximately 1.7 million inhabitants.^7^

The Calgary Zone Department of Medicine (DOM) comprises 431 primary members across 10 specialty sections^8^ and is embedded within the University of Calgary’s Cumming School of Medicine. Most departmental physicians hold academic appointments as university faculty and engage in clinical, research, education, and leadership activities. In 2019, DOM physicians cared for 15,292 hospitalized patients and provided 34,709 inpatient consults across the four urban adult hospitals.^8^ Prior to the pandemic, inpatient care for non-ventilated patients with severe respiratory infections was provided directly by General Internal Medicine and Pulmonary Medicine teaching teams comprising attending physicians with both inpatient and outpatient clinical practices, and variable participation among undergraduate and post-graduate clinical trainees at all levels (e.g., clinical clerks, PGY-1 junior residents, PGY-2-3 senior internal medicine residents, and PGY-4-5 GIM/Respirology subspecialty residents). These teams were complemented by allied healthcare providers including pharmacists, nurse practitioners, and rehabilitation services.

Responding to the COVID-19 pandemic, pre-existing provincial health system emergency operations were initialized including a provincial Emergency Central Command, five geographic Zone Emergency Operations Commands, and Site Command Posts at individual hospitals and healthcare facilities. The Calgary Zone pandemic response included four capacity stages, with corresponding expansions in acute care and critical care bed capacity (up to 2060 and 446 additional beds, respectively) (Carmella Steineke, Chair of Calgary Zone Capacity Planning Committee, personal communication) and cohorting of patients with suspected or confirmed COVID-19.

Physician workforce planning was delegated to Zone Clinical Department Heads, who were expected to address acute care demand surges while maintaining pre-existing acute care and ambulatory clinical services. In addition to the DOM, frontline acute care services most affected by the COVID-19 pandemic included Emergency Medicine, Critical Care, and inpatient Family Medicine services.

## INTERVENTION: MEDICAL EMERGENCY-PANDEMIC OPERATIONS COMMAND

**Figure 1:**
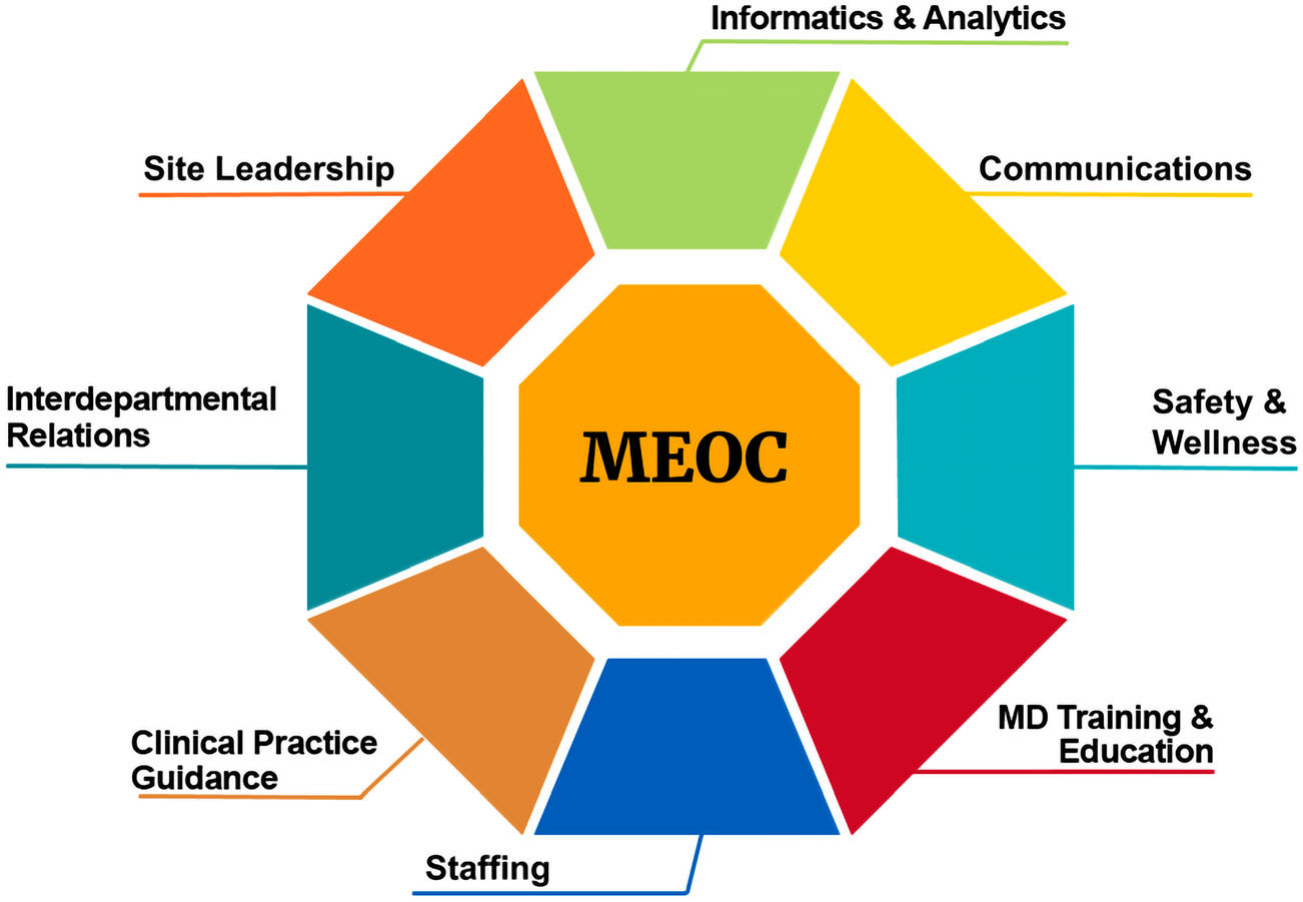
MEOC components.

Early Medical Emergency-Pandemic Operations Command (MEOC) planning brought together approximately 30 internal medicine physicians. There were seven key components of the MEOC Pandemic Plan, which we describe below and present in Figure 1 and Table 1. MEOC’s core mission was to provide *scalable, sustainable, and safe* COVID-19 patient care in the Calgary Zone. We include additional plan details in study supplement.

**Table 1:** Structure, Process, and Implementation Outcomes of MEOC Pandemic Plan by Key Component

	Structure	Process	Implementation outcome	Challenge
Staffing	•Scalable Pod structure (Pod leads/MDs) •Modified shift duration and rotation	•System for recruitment, onboarding, scheduling, and deployment of MDs	•1446 volunteers ○ 848 “practice-ready”* •93 MDs completed Pod shifts	•MD remuneration misaligned with staffing model •MDs with concurrent ambulatory duties
Interdepartmental relations	•MD liaisons: Critical Care, Emergency Medicine, Family Medicine, IPC •Resource bundles for inpatient care/discharge	•Workflow processes for admission and transfer •Protocols for clinical escalation/de-escalation	•6 clinical algorithms •New COVID liaison role •Daily care checklist	•Local processes limited standardization •Variable MD practice
Informatics and analytics	•Standardized admission order set •Clinical documentation templates in EMR •Embedded analysts	•Daily review of current and projected COVID-19 case volume •Dashboard monitoring of MD workload (for Pod deployment)	•319 uses of order set	•Some MDs reluctant to use electronic tools •Variation in clinical documentation across health professions
MD training and education	•PPE training modules •Hospital orientation •Simulation laboratories	•Mastery-based PPE training certification •Acute care shadow shifts for Pod MDs	•PPE training completed ○ 350 online ○ 227 in-person	•Limited shadow shifts on COVID-19 units due to low case volume
Clinical practice guidance	•COVID-19 management pathway •Participation on provincial COVID-19 scientific advisory group	•Synthesized evidence to inform clinical guidelines •Liaison with COVID-19 study investigators	•1118 visits to MEOC Resources guidelines page	•Rapidly emerging evidence on COVID-19 across many disciplines •Adherence to study protocols burdensome for MDs
Communications	•MEOC Resources website •MEOC Daily Bulletin •Online webinars^4^ •Office of CME&PD	•Bi-weekly synthesis of frontline feedback •General feedback via MEOC email address	•Outbound ○ 1501 website visits ○ 609 bulletin recipients ○ 2599 attended webinar •Inbound ○ 45 feedback forms	•Tracking and timely response to feedback •Rapidly changing clinical protocols
MD safety and wellness	•Liaison with provincial MD wellness groups •DOM MD Wellness Chair •Curated list of hospital logistical supports	•Dissemination of pandemic-related MD wellness resources •Psychological support for MDs •Consideration of health risk and inequities in staffing Pods	•50 visits to MEOC Resources wellness page**	•Exacerbation of inequities/stressors among DOM members

*DOM*, Department of Medicine; *EMR*, electronic medical record; *IPC*, Infection Prevention and Control; *MD*, physician; *MEOC*, Medical Emergency-Pandemic Operations Command; *PPE*, personal protective equipment.

*“Practice-ready” included actively licensed or retired physicians (see Table 2)

**Does not include visits to external links via MEOC Resources page

### MEOC COMPONENTS

#### Governance

**Figure 2:**
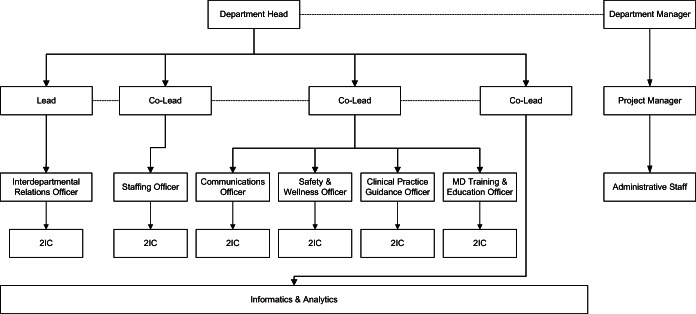
MEOC organizational chart. 2IC, second in command.

Adapting evidence from other health and humanitarian crises,^9^ MEOC created a governance model with a hierarchical command structure that included leadership and administrative teams, and seven sub-groups representing the plan’s key components (Fig. 2). In total, MEOC comprised 31 physicians, the DOM manager, the DOM project coordinator, and two clinical trainees with experience in military disaster response and logistics. Redundancy was built into each physician role to support continuity when MEOC members were deployed to clinical duties, ill, or required self-isolation.

#### Staffing

Acute care demand was expected to overwhelm the usual admitting services for patients with severe acute respiratory illness. MEOC developed a physician Pod team structure in which an experienced acute care physician (Pod Lead) would support up to three physicians with less acute care expertise (Pod MDs), each managing up to 15 inpatients (see eFig. 1). This structure could rapidly expand capacity and distribute acute care and subspecialty expertise across many patients. The model included a “COVID Liaison” (CL) physician, an indirect patient care role that coordinated patient admissions and transfers between COVID-19 units and the Emergency Department, Intensive Care Unit, or community hospitals. Due to concerns around safety and education-service balance, medical learners (both pre-and post-graduate) were not included in the initial workforce planning.

To reduce physician fatigue and risk of personal protective equipment (PPE) use errors, Pod teams were scheduled in eight-hour shifts for up to four consecutive days with at least three days off.^9, 10^ The MEOC Pandemic Plan incorporated a 25% back-up physician buffer for illness or isolation, based on experiences from other jurisdictions.^11–13^

MEOC developed a standardized recruitment process to rapidly deploy Pod physicians if inpatient demand surged. This process included confirming hospital privileges, PPE training, and an online shift scheduling tool. After iteration, these steps were systematized for management by administrative staff.

In Canada, most physicians are remunerated through one of two mutually exclusive payment models: fee-for-service or a contracted salary through an alternative relationship plan (ARP). In Alberta, physicians paid through fee-for-service billings were provided the option of remuneration by hourly sessional payment for shift work on COVID-19 teams, while contracted physicians continued with ARP remuneration.

#### Interdepartmental Relations

In partnership with other frontline clinical departments (Emergency Medicine, Critical Care, Family Medicine, Infection Prevention and Control (IPC)), MEOC developed standardized clinical care algorithms for efficient admission, transfer, and discharge of patients with COVID-19. Protocols guided care escalation or de-escalation and consultation of appropriate services (e.g., ICU vs. medical teams, Internal Medicine vs. Family Medicine). These processes aimed to support safe, rapid, and evidence-based decisions that were standardized across hospitals and easily used by both expert and non-expert acute care physicians. Finally, resource bundles integrated ambulatory services with inpatient care algorithms to ensure clinicians connected patients with post-hospital care and community resources (e.g., assisted isolation hotels for patients with insecure housing).

#### Informatics and Analytics

To facilitate efficient clinical documentation, MEOC instituted clinical decision support by creating standardized order sets for admission (eFig. 2) and hospital care (including clinical trials) within the local electronic medical record (EMR) (Sunrise Clinical Manager™, Allscripts LLC). Physicians were encouraged to adopt a hybrid documentation model that supplanted written clinical notes with a summative electronic discharge summary. To support this transition, MEOC members created standardized COVID-19 EMR templates.

MEOC estimated physician workload using hospital and Pod team censuses and COVID-19-related admission, transfer, and discharge activity through a newly created online dashboard (Tableau™, Tableau Software LLC). Furthermore, empirically derived epidemiologic COVID-19 models were used to project hospitalizations over a two-week horizon, allowing MEOC to match workforce supply and Pod team deployment across hospitals to existing and anticipated demand.

#### Physician Training and Education

MEOC medical education experts developed a three-part PPE course comprising: (1) an interactive mastery-based online certification module through the University of Calgary Office of Continuing Medical Education and Professional Development (CME&PD); (2) an in-person buddy-system session where participants practiced donning and doffing PPE supervised by IPC specialists; and (3) a high-fidelity patient care simulation session focused on appropriate PPE selection and use amidst additional physical, emotional, and cognitive stressors. In-person sessions were conducted in the University’s simulation laboratories.

MEOC also developed multiple supports to aid physicians deployed to unfamiliar hospitals or with less acute care expertise. These included hospital-specific orientation champions, videos, and logistic information (e.g., call and locker rooms, showers, nutrition); EMR tool training sessions; and quick reference guides for common acute care internal medicine presentations. Prior to scheduled shifts, Pod MDs were invited to complete hospital-specific orientation shifts shadowing Pod Leads on COVID-19 units or traditional inpatient medical services.

#### Clinical Practice Guidance

Rapidly emerging evidence was synthesized by MEOC’s Clinical Practice Guidance (CPG) working group to produce and update guideline documents, clinical pathways, and flow diagrams for frontline physicians. The CPG group also tracked and promoted COVID-19 clinical trials, providing patients with access to experimental therapy.

#### Communications

During health or humanitarian crises, clear, consistent, and bi-directional communication is critical to identify and address frontline problems.^14, 15^ Outbound communications were provided through a “MEOC Resources” website, a MEOC Daily Bulletin, and multiple online webinars. MEOC streamlined inbound communications from clinicians to MEOC leaders through a dedicated email address for general feedback and an online feedback form sent to frontline physicians after COVID-19 care shifts. The communications team thematically analyzed, aggregated, and presented reports summarizing front-line physician qualitative data bi-weekly to the MEOC executive, quality improvement teams, and site leaders to guide implementation and facilitate rapid response to frontline issues. MEOC tracked emerging issues via an online “issues-tracker” to organize and respond to implementation challenges.

#### Physician Safety and Wellness

The safety and wellness team curated an up-to-date list of physician logistic supports at each hospital, including nutrition services, rest areas, nap rooms, and scrub availability. They then engaged hospital leaders to identify and address deficiencies based on the projected number of physicians per site. Despite MEOC’s shift structure, nap rooms were made available for physicians at all times as recommended by MEOC’s physician wellness and sleep medicine experts.^16–19^

MEOC leveraged existing relationships with institutional and provincial physician wellness programs that developed pandemic-specific resources (e.g., informal physician peer-to-peer support, mental-health supports, and sleep strategies during COVID-19).^20^ These resources were curated on MEOC’s website, and physician wellness tips were included in MEOC Daily Bulletins. MEOC advocated for physician deployment plans that considered diversity, equity and inclusion, personal health issues (e.g., elderly physicians; physicians requiring immunosuppression), and life situations (e.g., two physician families with dependents).

## PANDEMIC PLAN IMPLEMENTATION

**Figure 3:**
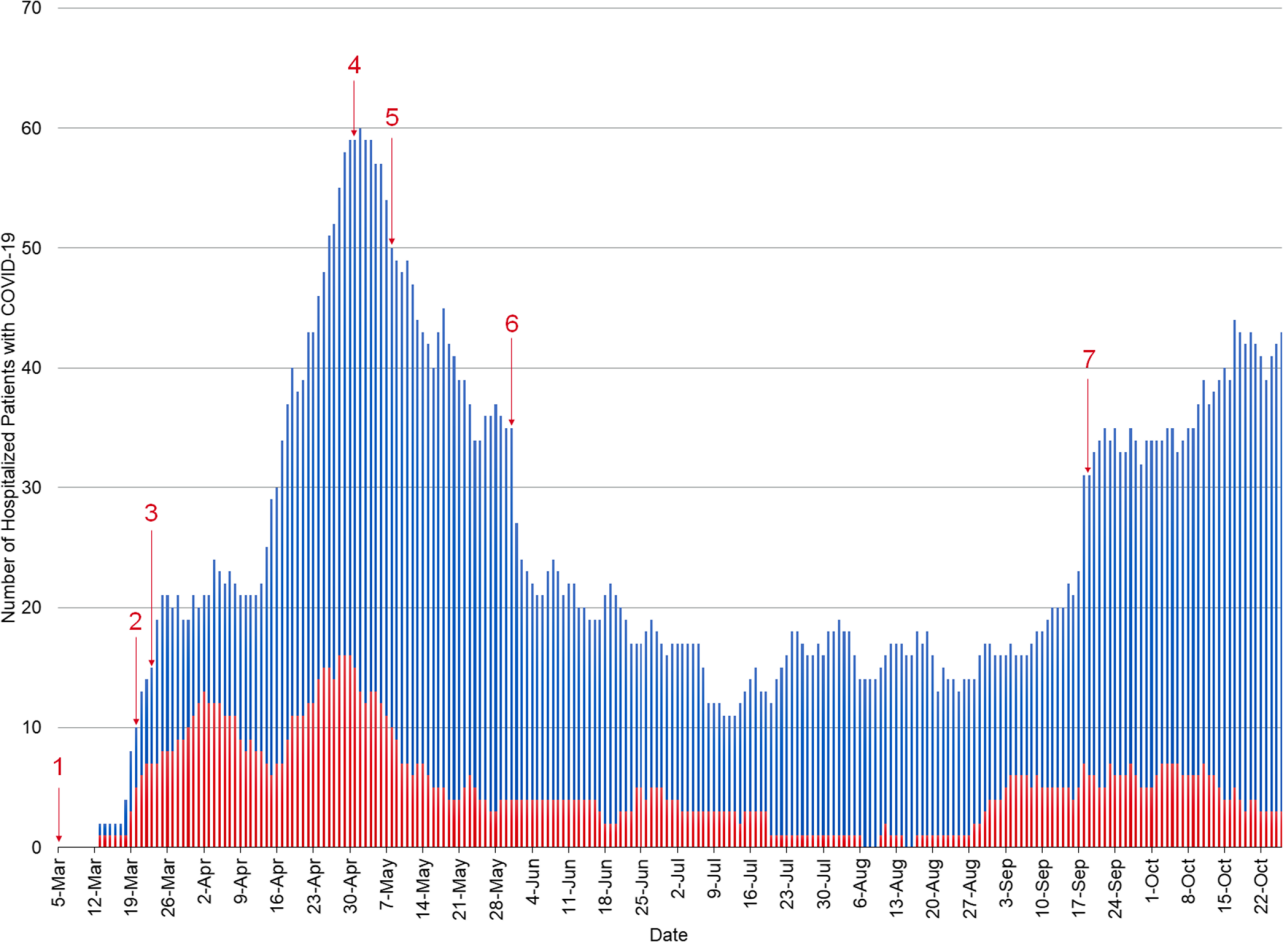
Implementation milestones for MEOC and number of admitted patients with COVID-19 in Calgary Zone from March 5 to October 26, 2020. Blue bars indicate total number of non-ventilated inpatients and red bars indicate number of patients in intensive care units.

From March 5 (first COVID-19 case in Alberta) to October 26, 427 and 88 COVID-19-infected patients were admitted to Calgary Zone acute care units and ICU, respectively (Fig. 3). Over the first six weeks (March 5–May 31, 2020), MEOC achieved several implementation outcomes (Table 1) with high physician engagement (Table 2). Following an initial webinar on March 27, 2020, in which MEOC leaders presented the Pandemic Plan and asked for physician volunteers, 1446 physicians registered to participate via an online recruitment tool, of whom 848 (58.7%) were in active clinical practice or recently retired and thus, eligible to provide medical care (Table 2). This represented 28% (495/1771) and 8.2% (215/2619) of licensed Calgary Zone specialists and family physicians respectively.^21^ The remaining 598 (41%) physician volunteers mostly comprised international medical graduates and other physicians who were ineligible to practice independently. Physician volunteer lists were distributed to respective clinical departments, and 234/848 (27.6%) physicians identified as potential Pod Leads or deemed most likely to integrate quickly onto COVID-19 Pod teams based on their clinical skills, training, and acute care experience completed the initial onboarding process. Notably, physician willingness to participate was unrelated to remuneration model. Within each clinical section, DOM leaders considered risk factors for poor COVID-19 outcomes among the physician workforce and accommodated these physicians within clinical schedules for essential non-COVID-19 services. Concurrently, university simulation laboratories were open seven days per week, allowing 227/234 (97%) physicians to complete all three PPE training components over three weeks. Many physicians reported that this PPE training was highly valuable.

**Table 2:** Physician Engagement

Physician recruitment and training	
Volunteers—1446* • 828 actively licensed • 20 retired Clinical departmental breakdown • Internal Medicine—187 • Family Medicine—215 • Other medical specialties (cardiac sciences, neurosciences, oncology)—44 • Other acute care (emergency, critical care, anesthesiology)—56 • Pediatrics—60 • Psychiatry—25 • Surgical specialities (surgery, obstetrics/gynecology)—114 • Diagnostic specialties (diagnostic imaging, pathology/lab)—9	
Communications	
MEOC Resources website visits (April 1 to May 11, 2020)—1501 (source: Google Analytics) MEOC webinars • Webinars hosted by University of Calgary Office of CME&PD ° March 27, 2020: 881 attendees ° April 3, 2020: 282/431 (65%) attendees (restricted to Department of Medicine) ° April 22, 2020: 848 attendees • Webinar hosted by University of Calgary O’Brien Institute for Public Health ° April 30, 2020: 588 attendees ▪ Facebook: 404 attendees; YouTube: 39 attendees; Zoom: 145 attendees ▪ Facebook geographic viewer breakdown: AB 76.2%; BC 15.6%; ON 4.94%; Montevideo, Uruguay 1.36%; California, USA 0.37%; QC 0.25%; MB 0.13%; Havana, Cuba 0.11%	

*CME&PD*, Continuing Medical Education and Professional Development; *MEOC*, Medical Emergency-Pandemic Operations Command

*570 ineligible for Alberta license (e.g., international medical graduates) or did not indicate status; 48 trainees

Designated COVID-19 Pods were activated at three adult hospitals. Sixty-five physicians worked as Pod Leads while 28 of 37 (75.7%) scheduled Pod MDs completed shifts. Fifty-three additional physicians were scheduled for back-up coverage, but not required. During implementation, MEOC received 45 physician feedback forms, providing actionable input regarding patient care processes, hospital resources for physicians, PPE procedures, and supply (see eTable 1). The COVID-19 admission order set was accessed 319 times and used for 65% of admissions.

Throughout implementation, outbound communications were frequent. Physicians accessed many resources; the MEOC Resources website received over 1500 visits from across Alberta. The MEOC Daily Bulletin was requested by clinical trainees, other clinical department heads, and zone health system leadership, reaching 609 individuals daily at peak distribution. Three MEOC webinars detailing the Pandemic Plan were attended by over 2000 physicians in total, and a public webinar describing MEOC activities was viewed by nearly 600 people (Table 2).

### MEOC PANDEMIC PLAN DE-ESCALATION AND RE-ESCALATION

In early May, epidemiologic data indicated that community and hospitalized COVID-19 cases were much lower than predicted (Fig. 3 and e Fig. 3). Updated projections suggesting a gradual increase in incident cases prompted several changes to MEOC’s structure and process. First, MEOC suspended Pod MD shifts and scheduling. Second, MEOC delegated decisions to maintain the Pod structure or use existing acute care services to manage patients with suspected or confirmed COVID-19 to each hospital’s DOM physician group. Third, given that COVID-19-related health system demand was expected to occur over many months, MEOC leadership was transitioned to pre-existing DOM administrative structures and traditional linkages to hospital operations. Hospital site leads, the clinical department head, department manager, and project coordinator assumed pandemic response leadership, while MEOC physician co-leads assumed consultative roles. Finally, MEOC embedded each Pandemic Plan component within existing DOM organizational structures, with continued involvement by many MEOC working group members.

In September 2020, rising COVID-19 case counts re-invigorated concerns about constrained hospital capacity (Fig. 3 and e Fig. 3). Provincial and Zone pandemic planning committees had been sustained, as had several MEOC components (e.g., EMR templates, wellness resources, outbound communications bulletins). In October, DOM leadership escalated many of these components and reactivated COVID-19 inpatient units staffed by Pod Leads.

### KEY IMPLEMENTATION INSIGHTS FROM MEOC

In addition to the MEOC Pandemic Plan’s core components, we identified three important considerations to enable implementation in other jurisdictions. First, many of MEOC’s successes depended upon a strong partnership between the health system and an academic institution. MEOC team members’ academic expertise included multi-specialty clinical care; health services, clinical, and policy research; disaster medicine; operations management; health analytics and informatics; medical education; physician wellness; and quality improvement. This collective expertise facilitated rapid synthesis of evidence and best practices to develop the MEOC Pandemic Plan. Finally, many MEOC members were contracted academic physicians with protected non-clinical time quickly re-allocated to MEOC plan development, implementation, and evaluation.

Strong clinical-academic ties supported real-time pandemic plan implementation in other ways. For example, MEOC leveraged many suspended university resources to host webinars, develop clinical tools, and train physicians. Once developed, DOM operations assumed control of these processes, ensuring that their sustainability was independent of temporarily available university resources. MEOC’s original plan did not incorporate clinical trainees; however, during de-escalation MEOC and Postgraduate Medical Education leaders formalized the inclusion of DOM residents in COVID-19 clinical services, thus expanding the skilled physician workforce.^23^ To date, clinical clerks have been excluded from any COVID-19 clinical care. These examples demonstrate a flexible partnership between a health system and an academic institution, beyond those well established for education and clinical research. Such collaborations align strongly with academic health systems’ mandates, can be implemented in other jurisdictions, and should be leveraged in future disaster responses.^22^

Second, MEOC’s early recruitment and demand-based staffing successes were facilitated by collaboration with other local groups, including the DOM administrative staff, other clinical departments, health system data analysts, and health informatics teams. Clearly identified leads assigned by their respective groups to liaise with MEOC leadership were key to these collaborations. As an ad hoc task force, MEOC did not immediately fit into complex pre-existing health system administrative structures. Although supported by DOM leadership, MEOC did not have a formal relationship with health system operations. This structural constraint complicated both escalation and de-escalation of the Pandemic Plan. MEOC’s transition to DOM leadership embedded the second wave’s re-escalation efforts within these complex health system administrative structures. For example, triggers to activate the Pod structure and deploy teams are now explicitly aligned with operational Zone-level targets, yet maintain flexibility for differential activation at each hospital based on site-specific patient demand.

Third, although the MEOC Pandemic Plan could scale to match acute care inpatient demand, it did not directly consider the workforce impact on ambulatory care, and the consequences of delayed non-COVID-19 ambulatory care. A dedicated zone ambulatory care planning group coordinated outpatient care for patients with non-COVID-19 illness considering public health measures and suspension of non-urgent clinical activities. MEOC included ambulatory care specialists in planning, engaged DOM section heads to identify “COVID-19 all-in” physicians whose ambulatory duties could be covered by other physicians, and aligned acute care follow-up processes with the ambulatory care group’s activities. However, at its core, the Pod-based staffing model depended on volunteerism by non-acute care physicians with concurrent ambulatory care responsibilities, neglect of which could further increase acute care demand. The evolving understanding of neglected chronic disease care during a pandemic,^23^ along with the transfer of workforce planning to DOM leadership, has enabled coordination of acute and ambulatory care strategies during the second wave.

## DISCUSSION

We describe a scalable, sustainable, and safe acute care COVID-19 pandemic physician workforce plan within an academic health system whose key components are transferable to other jurisdictions during healthcare crisis. Owing to strong collaboration among DOM members, this plan was novel in its breadth, its rapid development, and its implementation amidst an anticipated acute care demand surge. Hundreds of physicians participated in the MEOC Pandemic Plan’s design, implementation, and dissemination over a short time period, indicating it was perceived as urgent and highly relevant. To our knowledge, we are the first to report (although not to design) a COVID-19 physician staffing pandemic response that incorporates early implementation data and other important aspects of acute care delivery during a health crisis.

Despite rapid expansion of COVID-19-related literature, limited data exist to guide specific health system adaptations to the pandemic. In critical care settings, published reports have outlined disaster response strategies, including team-based models that incorporate non-expert physicians under supervision of trained physicians,^24–27^ adequate PPE supply and training,^15, 24, 26, 28–30^ and clear algorithms for efficient evidence-based clinical care.^24^ Outside of critical care, recent reports describe COVID-19 pandemic plans within academic health systems with high-level staffing model descriptions in the context of broader care delivery strategies.^28–30^

The MEOC Pandemic Plan shares many structures and processes with these published plans, thus supporting its implementation. However, unlike previous reports we include specific details regarding the governance, staffing, interdepartmental relations, informatics and analytics, physician training, clinical practice support, communications, and physician safety and wellness plan. Many of the pandemic plan’s core components are transferrable to other institutions facing the COVID-19 pandemic: physician engagement/volunteerism, scalable training capacity, and a clinical care model that has safety, wellness, and communication as key enablers. Moreover, we present early implementation data demonstrating high physician engagement as a precursor of longer-term effectiveness. These data provide valuable insights supporting the MEOC Plan’s iterative improvement and highlighting key considerations for other urban academic health systems and healthcare crises.

This study has important limitations. First, it is a single-site description, and its findings may not directly apply to other jurisdictions. However, MEOC’s purpose was to develop and refine a departmental pandemic response. In this manuscript, we aim to identify several components of a pandemic plan transferrable to other health systems and important related considerations. Second, the early trajectory of COVID-19 in Calgary limited our ability to evaluate MEOC’s Plan amidst high clinical demand. Unexpectedly low hospital volumes led to MEOC’s relatively abrupt de-escalation and administrative handoff. However, given the current resurgence in COVID-19 cases, we believe this preliminary description highlights challenges that might arise during larger scale plan activation. Finally, we used readily available data sources and did not include patient outcomes such as ICU transfer or mortality rates, nor clinician outcomes such as clinician COVID-19 infection rates. However, our findings provide a window into stakeholder engagement as an antecedent to successful implementation of a comprehensive physician workforce plan. Future work should explore additional patient and physician outcomes related to this pandemic response.

## CONCLUSION

The MEOC Pandemic Plan is a scalable, sustainable, and safe pandemic physician workforce plan rapidly co-developed within an academic department of medicine in partnership with a provincial health system. MEOC leveraged existing expertise and relationships with an academic institution to successfully develop, implement, and refine a comprehensive pandemic plan using knowledge from other global health and disaster medicine contexts. Although successful public health measures mitigated the need for large-scale plan activation during the pandemic’s first wave, its early implementation revealed broad multispecialty physician engagement and lessons to improve re-escalation efforts as the pandemic evolves. Our description highlights learnings over an accelerated design and implementation period and can guide other institutions currently responding to COVID-19 as well as future pandemics or health emergencies.

## Supplementary Information

ESM 1 (DOCX 2459 kb)
