# Supplementary material for: Description of a Multi-faceted COVID-19 Pandemic Physician Workforce Plan at a Multi-site Academic Health System
Source: J Gen Intern Med. 2021 Feb 9;36(5):1310–8. doi: 10.1007/s11606-020-06543-1 (PMC7872510; doi:10.1007/s11606-020-06543-1)
Supplement: Supplementary file 1 — (DOCX 2459 kb) [file 11606_2020_6543_MOESM1_ESM.docx]

## Description of a multi-faceted COVID-19 pandemic physician workforce plan at a multi-site academic health system

Supplementary Material

# Intervention: Medical Emergency-Pandemic Operations Command (MEOC)

## MEOC Components

## Staffing

There were several considerations in developing an acute care staffing model for the surge of patients with COVID-19. It was expected that the physician supply from traditional acute care services (i.e., GIM, respirology, nephrology, hematology) would be inadequate to respond to a large acute care demand; thus, there was a high likelihood that physicians with less acute care expertise (i.e., endocrinology, rheumatology, dermatology) or from outside the Department of Medicine (e.g. surgery, neurology, and pediatrics) would need to provide direct care for acutely ill patients with respiratory failure.

### Physician Pod Structure

A Pod team structure was developed in which an experienced acute care internal medicine physician (Pod Lead) would be partnered with up to three non-acute care or non-internal medicine physicians (Pod MD) to provide clinical support and co-management as needed. Furthermore, the number of patients managed by an individual Pod MD was limited to 15, recognizing variable comfort managing this patient population and additional time required to safely use PPE. This structure allowed the distribution of acute care expertise across a larger number of inpatients and allowed Pod MDs to provide consultation to other Pod team members within their areas of expertise. Additionally, predetermined schedules allowed one to three Pod MDs to be deployed as necessitated by patient demand (see eFigure 1).

The model also included a “COVID Liaison” (CL) to manage patient admission and transfer from the Emergency Department, Intensive Care Unit or community hospitals. By dedicating an additional physician to this task, risks of repeated PPE exchanges or movement of Pod physicians between dedicated COVID-19 units and other parts of the hospital could be minimized. Importantly, this role did not require in-person patient assessment and could thus be performed by physicians with acute care experience who could not otherwise provide direct COVID-19 care due to advanced age, chronic illness or furlough.

Medical learners (pre- and post-graduate) were not included in the initial workforce planning due to their critical role in traditional hospital-based services and concerns about balancing education and service. However, MEOC and Postgraduate Medical Education leaders regularly communicated to identify strategies for involving the resident workforce in the care of patients with COVID-19 if required.

### Physician Shift Structure

Evidence from other epidemics indicated that shift length and rotation structure had meaningful impacts on physician fatigue and risk of PPE errors.(1,2) Thus, Pod teams were scheduled in eight-hour shifts for up to four consecutive days with at least three days off in between; the latter structure required two teams per eight-hour shift each week. Third, the model incorporated a 25% buffer of backup physicians based on epidemiologic projections and experience from other jurisdictions.(3–5) Locally, the need for this backup schedule was highlighted by delayed diagnosis of COVID-19 in a single inpatient, resulting in unprotected exposure and isolation of approximately 40 healthcare workers (including six physicians) at one hospital.

###

### Physician Recruitment and Onboarding

Finally, a standardized recruitment and onboarding process was established to rapidly identify and deploy Pod team members if inpatient demand surged. Process steps included: 1) a broadly disseminated online webinar to introduce the physician workforce plan and call for volunteers; 2) a Zone-wide online physician registration form to facilitate recruitment; 3) activation of appropriate hospital privileges; 4) PPE training (see *Physician Training and Education* below), and; 5) an online shift scheduling tool in which physicians could provide their availability. In parallel, physicians who volunteered were vetted by their own clinical department heads to ensure that pre-existing essential clinical services within each department were adequately staffed. The recruitment, onboarding, scheduling and deployment steps were systematized and managed by administrative staff to offload MEOC members who would be required to provide clinical care.

### Remuneration

Physicians in Alberta are remunerated through one of two mutually exclusive models: fee-for-service billings based on a fee schedule managed by the Provincial Ministry of Health or contracted alternative relationship plans (ARPs) for which clinical deliverables are based on the same fee schedule. The Department of Medicine comprised 42% fee-for-service and 58% salaried physicians. Neither remuneration structure aligned with the proposed shift work model for inpatient care, nor were there provisions for uncertainty in patient demand that would dictate deployment of physicians. Following negotiations between the Ministry of Health, AHS Calgary Zone leadership and MEOC leadership, an optional hourly sessional payment was established for fee-for-service physicians performing shift work on COVID-19 teams. No remuneration was provided for scheduled back-up physicians who did not provide clinical services. Contracted physicians continued to be remunerated via the ARP.

## Interdepartmental Relations

Anticipating high patient volumes, MEOC members engaged other critical frontline clinical departments (Emergency Medicine, Critical Care, Family Medicine, Infection Prevention & Control (IPC)) to standardize and streamline care transitions between clinical services. These included algorithms for efficient admission, transfer and discharge of patients with COVID-19. Anticipating critical care beds and ventilator capacity would be an important and limited acute care resource in this pandemic, MEOC created standardized escalation and de-escalation protocols for patients who either clinically deteriorated on a hospital COVID-19 unit requiring transfer to the intensive care unit (ICU) or recovered and were successfully extubated and required transfer from the ICU to a COVID unit. Finally, MEOC developed clinical algorithms, data collection forms and guidelines for consultation of appropriate services (e.g. ICU vs medical teams, Internal Medicine vs. Family Medicine). These processes aimed to support safe and protocolized rapid decision making while following emerging evidence and best practice.

Through the collaboration of multiple stakeholders in the Calgary Zone, several resources were developed to optimize outpatient primary and specialty care management of recently discharged patients with COVID-19, and improve access for those with non-COVID-19 illness. Resource bundles were created to integrate these ambulatory services with inpatient care algorithms and also to ensure clinicians could connect patients with community resources such as housing or assisted isolation hotels for patients with insecure housing.

## Informatics and Analytics

The local electronic medical record (Sunrise Clinical Manager™, Allscripts LLC; SCM) was leveraged to facilitate efficient delivery of care and documentation during a large patient surge. Clinical decision support was instituted through the creation of standardized order sets for admission (eFigure 2) and hospital care (including within clinical trials). Pre-selected orders indicated early warning thresholds to automate the escalation of concerns to physician teams. Given frequent shift changes and anticipated high patient turnover, physicians were encouraged to adopt a hybrid documentation model in which conventional clinical progress notes were supplanted by a summative discharge summary. To support this transition, MEOC created clinical documentation templates for admission and discharge through associated EMR tools (Notecetera™, Allscripts LLC). To accommodate the new staffing model MEOC implemented clinical team structures within SCM to facilitate patient admission and transfer to and from Pods. Physician workload estimates were estimated by capturing per hospital, per pod-team census, admission, transfer and discharge activity in an online dashboard (Tableau™, Tableau Software LLC). In this way, MEOC could rapidly match workforce supply to existing and anticipated demand and adjust Pod teams across different hospitals. The inpatient census and empirically derived epidemiologic models were used as a basis for two-week staffing projections to plan for Pod physician deployment.

## Physician Training and Education

The estimated surge in hospitalized patients with COVID-19 raised specific challenges for physician training and education. First, there would be a large expansion of the frontline physician workforce, all of whom would be at risk of exposure to COVID-19. Second, physicians could potentially be deployed to unfamiliar hospitals, necessitating site-specific orientation information and onboarding.

### PPE Training and Simulation

To promote physician safety, there was an urgent need to provide faculty development in personal protective equipment (PPE) to all physicians. A three-part course was developed by MEOC medical education experts, comprising: an interactive mastery-based online certification module housed through the University of Calgary Office of Continuing Medical Education and Professional Development (CME&PD); an in-person buddy-system practice session in which participants practiced donning and doffing with the use of checklists under supervision of an IPC specialist; and a patient care simulation session focused on correct use and selection of PPE in the context of additional physical, emotional, and cognitive stressors. All in-person sessions were conducted in the University’s simulation laboratory (re-opened despite facility closures with permission from the Dean) and respected physical distancing and other infection prevention measures.

### Orientation and Hospital Onboarding

Orientation information was developed in collaboration with a medical education expert at each of the four urban hospitals with the engagement of content-area experts as needed. Key topic areas covered included: hospital-specific orientation videos; logistical information (e.g., parking, card access, locations of on-call suites, etc.); electronic medical record (EMR) training guides; and quick reference guides for common acute care internal medicine presentations. Prior to scheduled shifts, Pod MDs were also provided the opportunity to gain experience by shadowing acute care leads on COVID-19 units or traditional inpatient medical services.

## Clinical Practice Guidance

All physicians providing care to patients with COVID-19 would be required to digest and synthesize a rapidly expanding evidence base. To provide a consistent and standardized evidence-based approach to treatment across the Calgary zone, MEOC established a Clinical Practice Guidance (CPG) working group that comprised Internal Medicine specialists from multiple disciplines. The working group’s specific objectives were to review and evaluate emerging evidence and develop guideline documents or clinical pathways to support frontline clinical care. These efforts were complemented and supported by AHS’ Scientific Advisory Group which provided rapid literature reviews in response to specific clinical questions. The CPG working group also aimed to support COVID-19 research efforts by tracking and promoting clinical trials and patient enrollment processes to physicians. This was a critical role as the only access for patients to experimental therapies was through clinical trials

## Communications

During health or human crises, clear, consistent and bi-directional communication is of utmost importance to keep physicians informed and to identify problems experienced by frontline providers;(6) thus, the MEOC Pandemic Plan included a strategy for both outbound and inbound communications.

### Outbound Communications

Outbound communications were provided through a ‘MEOC Resources’ section of the Department of Medicine’s website (the MEOC Resources website) and a standardized ‘MEOC Daily Bulletin’. Multiple online webinars were hosted in partnership with the University’s Office of CME&PD and sent ad hoc email distributions of news/announcements to department members. The aims were: 1) to rapidly distribute information about the extent and evolution of the pandemic to members of the Department of Medicine; 2) to solicit physician volunteers for Pod-based care of COVID-19 patients; and 3) to share training opportunities, educational materials and clinical algorithms developed by AHS or MEOC working groups.

### Inbound Communications

A streamlined process for department members to communicate with the MEOC leadership team included a dedicated email address for general queries and feedback on the MEOC Pandemic Plan, which the Communications team collated and directed to the appropriate team members; more urgent concerns requiring rapid escalation or process changes could be communicated in this way. Additionally, an online feedback form was distributed by email to actively solicit feedback from frontline physicians following COVID-19 call shifts. The free-text form addressed four main domains: aspects of their shift that worked well, what didn’t work well, unexpected challenges, and suggestions for improvement. The Communications team thematically analyzed, aggregated and presented reports summarizing front-line physician qualitative data bi-weekly to MEOC leadership, quality improvement teams, and site leaders to address knowledge gaps and refine care processes.

## Physician Safety and Wellness

The MEOC Pandemic Plan embedded physician safety and wellness within all processes by identifying existing and emerging best practises to protect physicians and support wellness. Strategies to enhance safety included rigorous training in the use of PPE, the staffing model and coordinated opportunities for N95 mask fit testing. To ensure logistic support, MEOC and leadership at each acute care hospital site itemized existing nutrition services, showers, change rooms and lockers, call rooms, rest areas and nap rooms, and availability of scrubs. Each site then strategized solutions to address deficiencies based on needs proportionate to the number of physicians deployed. An up-to-date list of logistic supports was provided to redeployed physicians. Despite MEOC’s shift structure, nap rooms were made available for physicians at all times as recommended by MEOC’s physician wellness and sleep medicine experts.(7–10)

An existing culture of physician wellness facilitated a rapid response to the psychological distress experienced by physicians due to the pandemic. We leveraged existing relationships with institutional and Provincial wellness programs, which had pivoted their focus to develop resources specific to pandemic-related threats to physician wellness (e.g., informal physician peer-to-peer support, mental-health supports and sleep strategies during COVID-19).(11) Information on support resources were curated and presented on the MEOC Resources website and a physician wellness tip was included in MEOC Daily Bulletins. consider physician deployment through a lens of diversity, equity and inclusion, considering personal health issues (e.g., elderly physicians; physicians requiring immunosuppression) and life situations (e.g., two physician families with dependents).

# References

1. National Institute for Occupational Safety and Health, Occupational Safety and Health Association. Preventing worker fatigue among Ebola healthcare workers and responders. Washington (DC): National Institute for Occupational Safety and Health; 2015 p. 1–4.

2. Dai H, Milkman KL, Hofmann DA, Staats BR. The Impact of Time at Work and Time Off From Work on Rule Compliance: The Case of Hand Hygiene in Health Care. J Appl Psychol. 2015;100(3):846–62.

3. Barrett ES, Horton DB, Roy J, Gennaro ML, Brooks A, Tischfield J, et al. Prevalence of SARS-CoV-2 infection in previously undiagnosed health care workers at the onset of the U.S. COVID-19 epidemic [Internet]. Infectious Diseases (except HIV/AIDS); 2020 Apr [cited 2020 Jun 15]. Available from: http://medrxiv.org/lookup/doi/10.1101/2020.04.20.20072470

4. Bellizzi S, Fiamma M, Arru L, Farina G, Manca A. COVID-19: The daunting experience of healthcare workers in Sardinia, Italy. Infect Control Hosp Epidemiol. 2020 Apr 20;1–2.

5. Chirico F, Nucera G, Magnavita N. COVID-19: Protecting Healthcare Workers is a priority. Infect Control Hosp Epidemiol. 2020 Apr 17;1–1.

6. Dodgen DW. Office of the Assistant Secretary for Preparedness and Response: (658332011-001) [Internet]. American Psychological Association; 2011 [cited 2020 Jun 15]. Available from: http://doi.apa.org/get-pe-doi.cfm?doi=10.1037/e658332011-001

7. Brooks A, Lack L. A brief afternoon nap following nocturnal sleep restriction: which nap duration is most recuperative? Sleep. 2006 Jun;29(6):831–40.

8. Mednick SC, Cai DJ, Kanady J, Drummond SPA. Comparing the benefits of caffeine, naps and placebo on verbal, motor and perceptual memory. Behav Brain Res. 2008 Nov 3;193(1):79–86.

9. Rajaratnam SMW, Howard ME, Grunstein RR. Sleep loss and circadian disruption in shift work: health burden and management. Med J Aust. 2013 Oct 21;199(8):S11-15.

10. Rosekind MR, Smith RM, Miller DL, Co EL, Gregory KB, Webbon LL, et al. Alertness management: strategic naps in operational settings. J Sleep Res. 1995 Dec;4(S2):62–6.

11. Well Doc Alberta COVID-19 Physician Wellness Resources [Internet]. 2020 [cited 2020 Jun 15]. Available from: https://www.welldocalberta.org/covid-19-updates

## eTable 1: Summary of Frontline Feedback

| Theme | Feedback Items |
| --- | --- |
| Infection Prevention & Control | - Availability of PPE - Isolation procedures - Appropriate PPE use by healthcare workers and hospital staff |
| Patient Flow | - Admission processes - Discharge algorithms - Family visitation rules |
| COVID Pager | - Clarification of appropriate calls to COVID-19 pager |
| Physician Wellness | - Stigma of working on COVID-19 designated unit - Lack of spaces for breaks, nutrition - Access to call rooms |
| Staffing Model | - Value of shadow shifts for non-acute care physicians - Remuneration challenges with staffing model |

## eFigure 1: MEOC Staffing Model


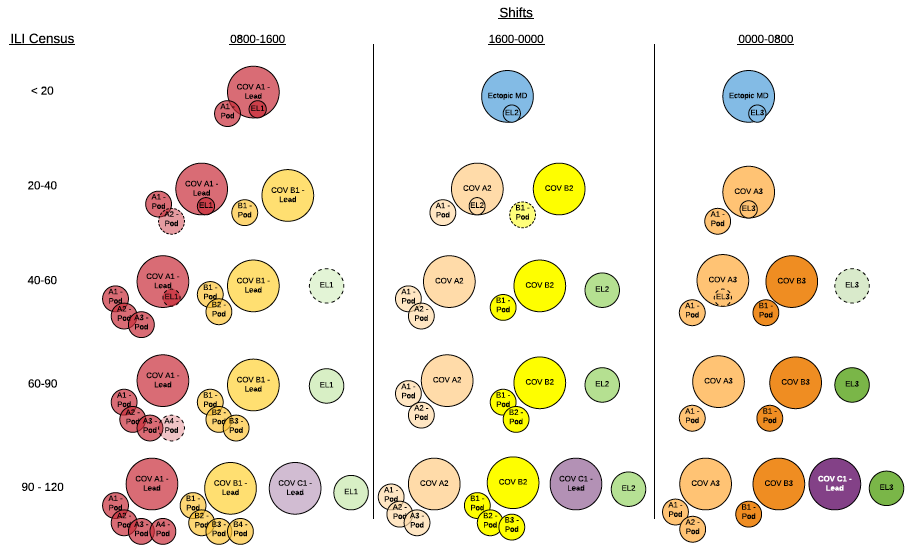


ILI – Influenza-like illness; EL – Emergency (COVID) Liaison; MD – physician; MEOC – Medical Emergency-Pandemic Operations Command

## eFigure 2: Order Set Screenshots from Electronic Medical Record


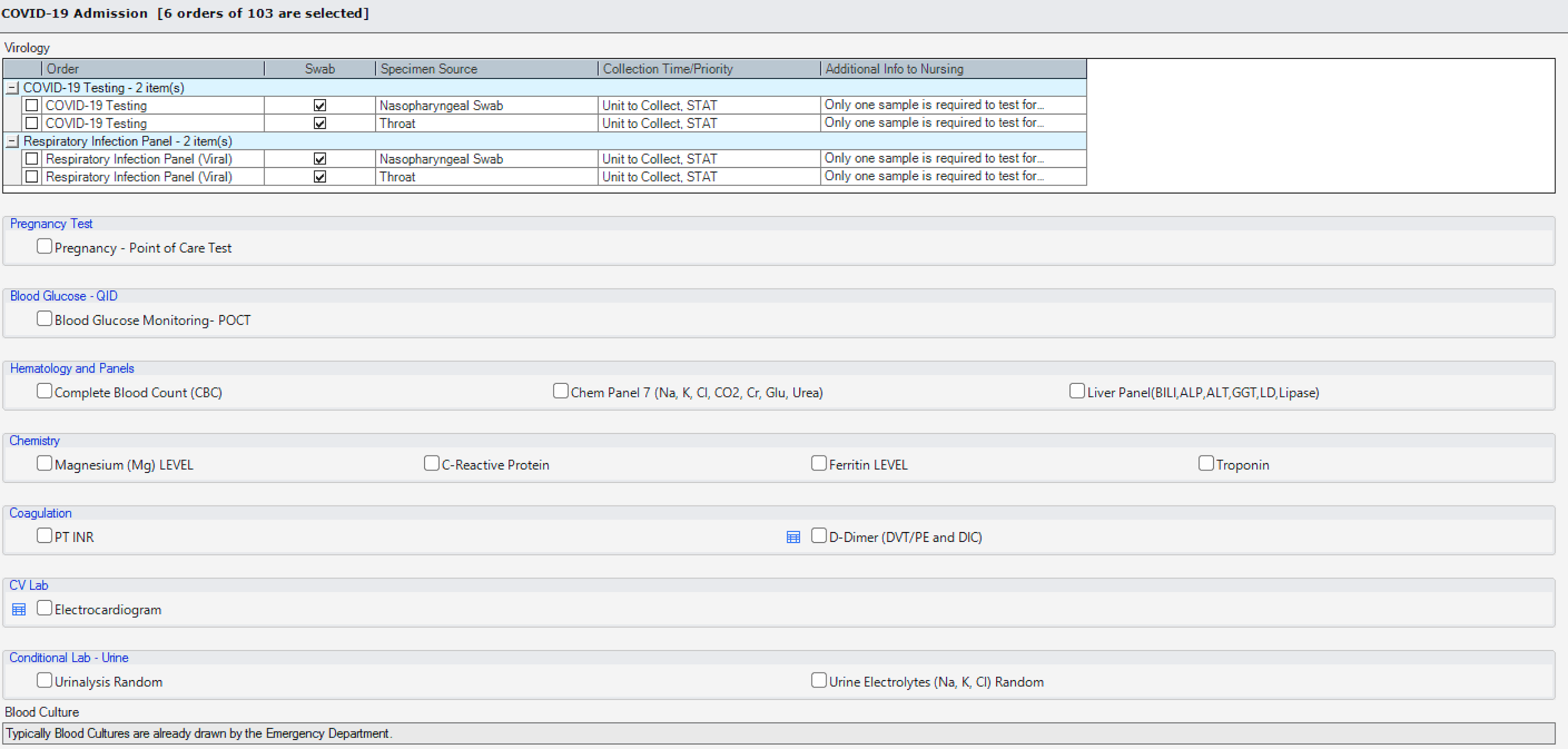

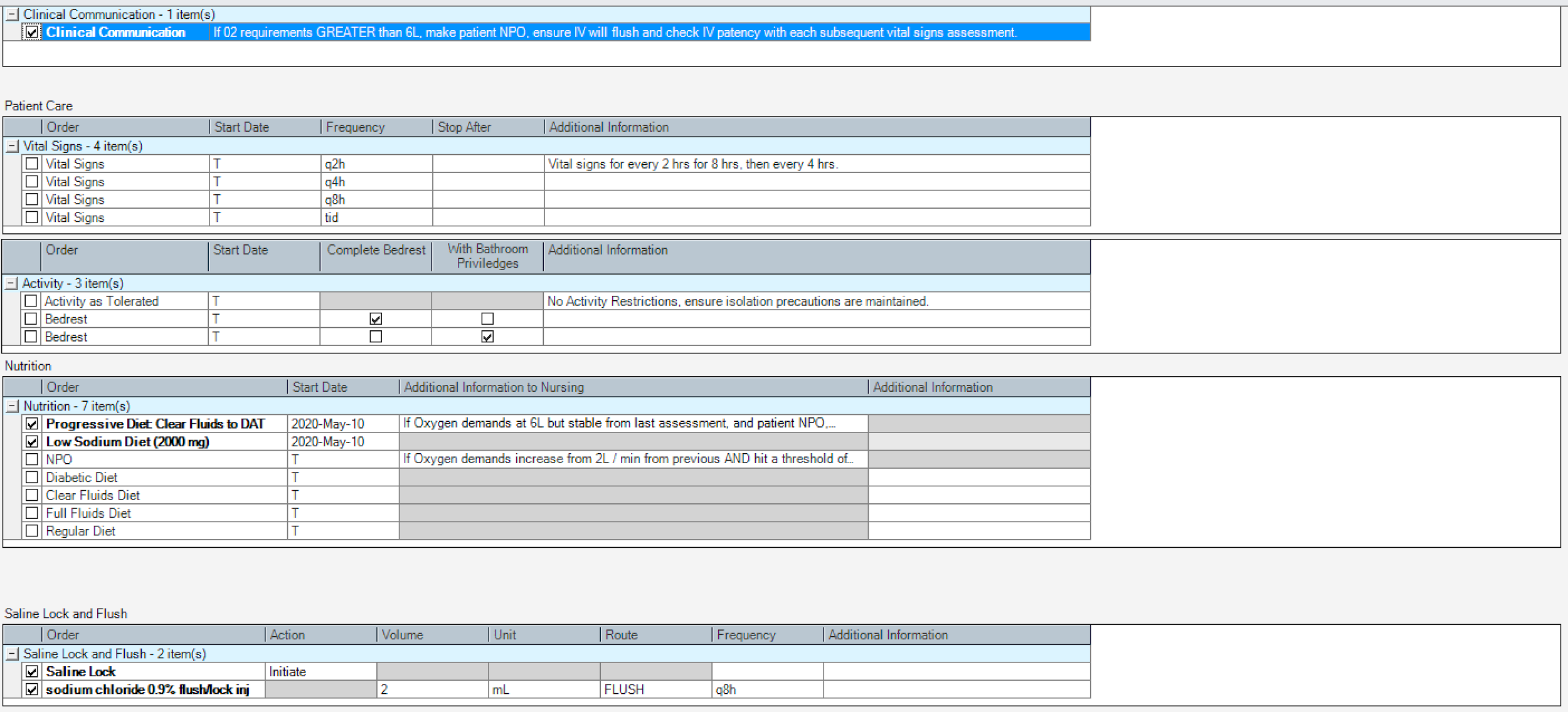

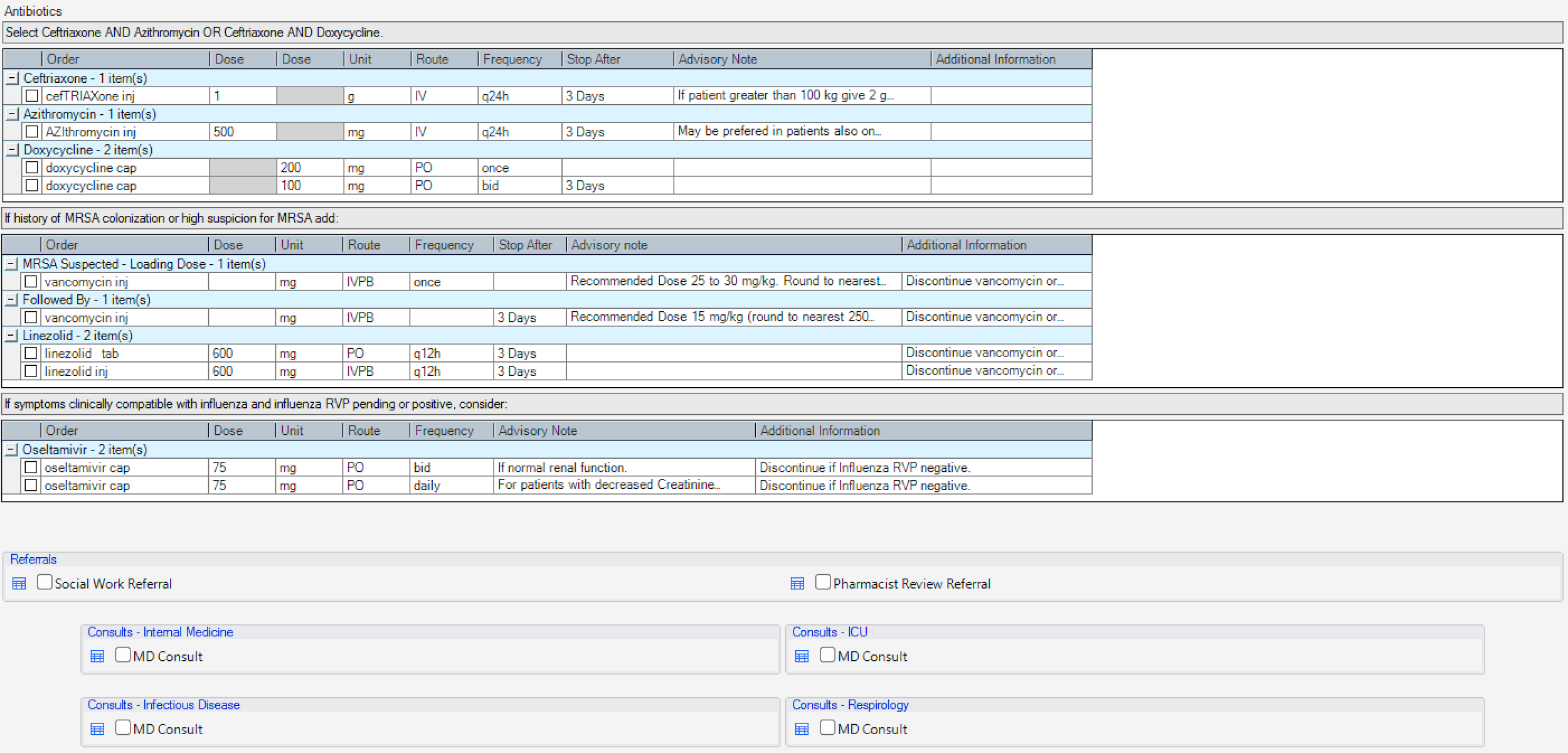

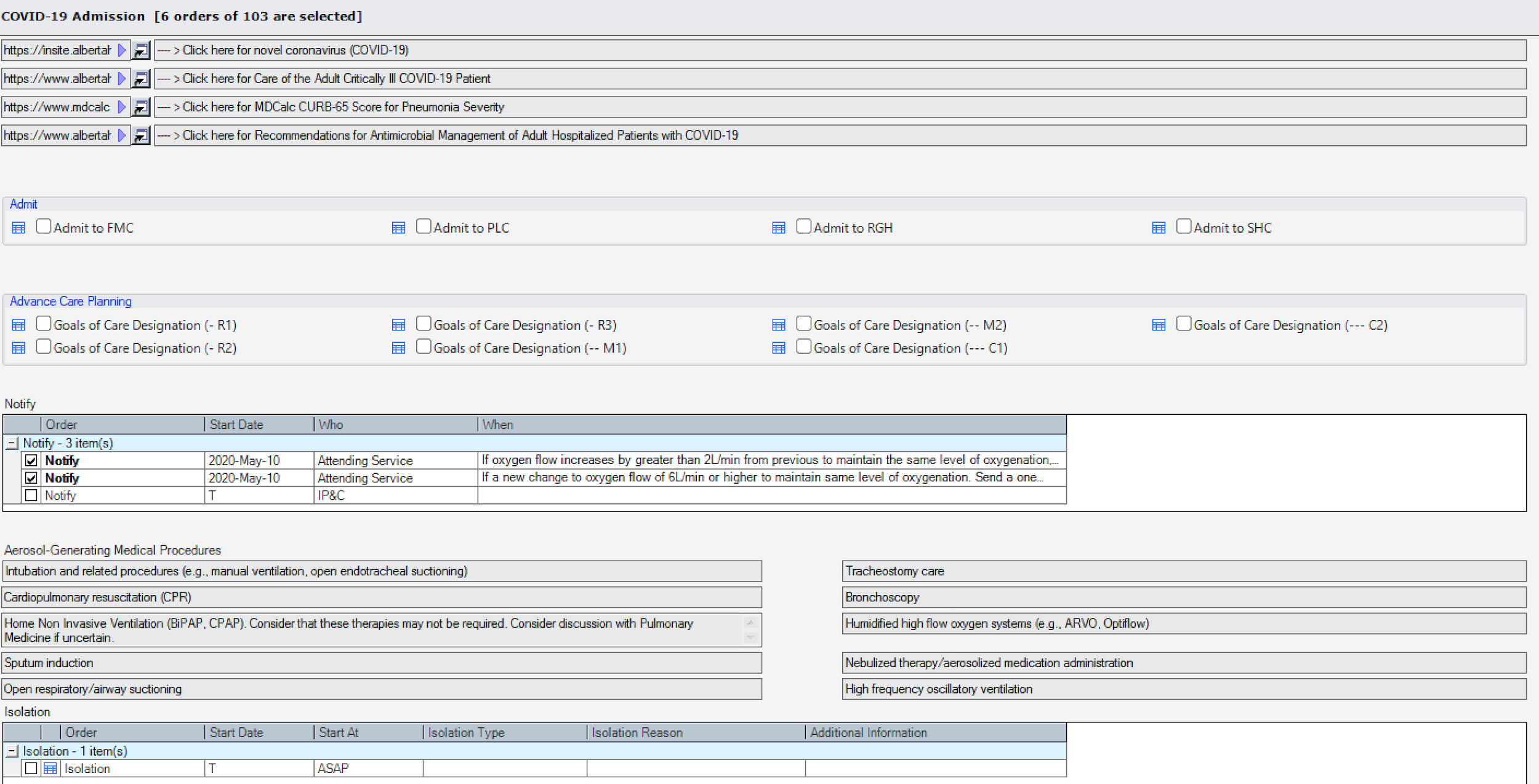


**eFigure 3:** Implementation milestones for MEOC and number of admitted patients with COVID-19 in Alberta from March 5-October 26, 2020.


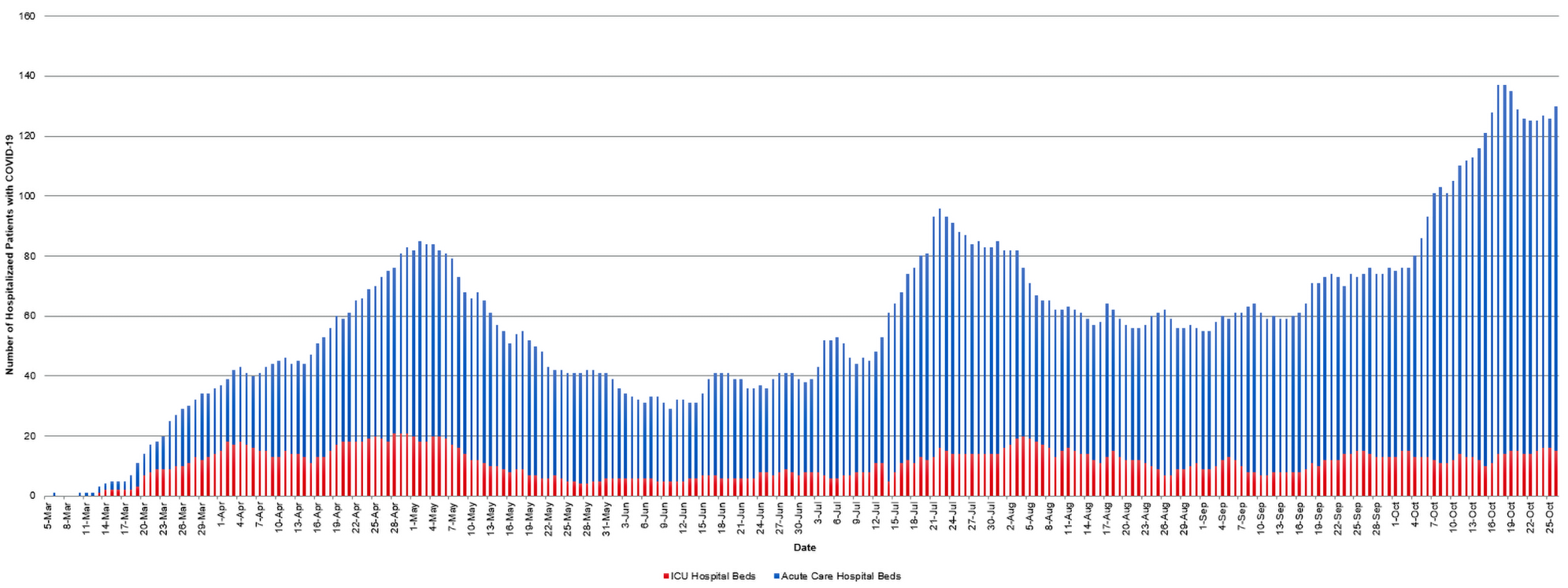


Blue bars indicate total number of non-ventilated inpatients and red bars indicate number of patients in intensive care units.
